# Occupational exposure factors for mental and behavioral disorders at work: The FOREC thesaurus

**DOI:** 10.1371/journal.pone.0198719

**Published:** 2018-06-21

**Authors:** Alain Chamoux, Céline Lambert, Audrey Vilmant, Charlotte Lanhers, Raymond Agius, Mounir Boutaleb, Vincent Bonneterre, Geraldine Naughton, Bruno Pereira, Khalid Djeriri, Eric Ben-Brik, Christine Breton, Caroline De Clavière, Corinne Letheux, Anne-Gaëlle Paolillo, Madeleine Valenty, Odile Vandenberghe, Marie-Pierre Aeschlimann, Gérard Lasfargues, Members of the RNV3P, Francois-Xavier Lesage, Frédéric Dutheil

**Affiliations:** 1 CHU Clermont-Ferrand, University Hospital of Clermont-Ferrand, Occupational Medicine, Clermont-Ferrand, France; 2 CHU Clermont-Ferrand, University Hospital of Clermont-Ferrand, Clinical Research and Innovation Direction, Clermont-Ferrand, France; 3 University of Manchester, Institute of Population Health, Centre for Occupational and Environmental Health, Centre for Epidemiology, Manchester, United Kingdom; 4 CHU Grenoble, University Hospital of Grenoble, Occupational Medicine, Grenoble, France; 5 Australian Catholic University, Faculty of Health, Melbourne, Victoria, Australia; 6 CHU Poitiers, University Hospital of Poitiers, Occupational Medicine, Poitiers, France; 7 Regional Health Insurance Fund of Ile de France (CRAMIF), Prevention of Occupational Risks, Paris, France; 8 CHU Créteil, University Hospital of Créteil, Occupational Medicine, Créteil, France; 9 Interdepartmental Center of Health and Occupational Medicine in Factories (Cisme), Paris, France; 10 CHU Garches, University Hospital of Garches, Occupational Medicine, Garches, France; 11 The French Institute for Public Health Surveillance (InVS), Paris, France; 12 The French National Health Insurance System (CNAM), Paris, France; 13 CHU Toulouse, University Hospital of Toulouse, Occupational Medicine, Toulouse, France; 14 Environmental and Occupational Health and Safety (ANSES), Maisons-Alfort, France; 15 University of Montpellier, CHU, Montpellier, France; 16 Epsylon, Univ Paul Valery Montpellier 3, Montpellier, France; 17 Université Clermont Auvergne, CNRS, LaPSCo, Physiological and Psychosocial Stress, Clermont-Ferrand, France; 18 University Hospital of Clermont-Ferrand, Occupational and Preventive Medicine, WittyFit, Clermont-Ferrand, France; University Antwerp, BELGIUM

## Abstract

**Background:**

Mental disorders in the workplace are a major public health problem. Knowledge of the impact of the psychosocial work environment on mental and behavioral disorders can assist occupational physicians in the identification and description of occupational risk situations, and help to define priority actions. However, no classification for occupational exposure factors is currently available. We aimed to build a thesaurus of “Organizational, Relational, Ethical and other Contributing Factors” (FOREC) linked with the onset of mental and behavioral disorders.

**Methods:**

The French Agency for Food, Environmental and Occupational Health and Safety (ANSES) initiated and supervised a multidisciplinary working group consisting of the representatives of the main French occupational and public health actors. All decisions were accepted on a consensus basis. This collaborative work led to the classification of occupational exposure factors for mental and behavioral disorders in the workplace. To test this thesaurus in clinical practice, a French multicenter study was implemented. Patients were workers referred to the Occupational Disease Centers for mental health issues at work. Factors contributing to mental and behavioral disorders among workers were identified and coded retrospectively from the worker’s point of view using the FOREC thesaurus.

**Results:**

We recruited 323 workers, aged 44.9±9.2 years, of which 31.3% were men. The most commonly encountered disorders were generalized anxiety disorders (106 workers, 32.8%) and moderate depressive episodes (86 workers, 26.7%). We identified 1357 factors, i.e. an average of 4.2 factors per worker. Among them, 575 (42.4%) were relational and 515 (37.9%) were organizational. All factors identified during consultations were described in the thesaurus.

**Conclusions:**

We built the first thesaurus of “Organizational, Relational, Ethical and other Contributing Factors” (FOREC) that may help to generate profiles of mental and behavioral disorders at work. Encoding and describing these exposure factors, as well as using a worldwide standardized and shared terminology, will help to identify specific workplace prevention programs.

## Introduction

Mental disorders are major public health problems in the workplace [1]. Work stressors, such as psychological and organizational demands (e.g. work pace, time pressure, complexity of work and conflicting tasks [2]), have an impact on common mental disorders [3]. Mental disorders increase business and social costs due to losses in productivity [4,5,6,7], sick leave [8,9] and staff turnover (Ref. needed). Mental disorders are also associated with morbidity and mortality [10,11,12]. For these reasons, work-related mental disorders constitute an important public health challenge.

Since 2000, the French National Occupational Diseases Monitoring and Prevention Network (RNV3P) has been gradually expanded in metropolitan areas of France. This network collects data, in a sustainable and coordinated way, from Occupational Disease Centers in 32 university hospitals and about ten occupational health services. The initiative targets the improvement and harmonization of practices for the diagnosis of work-related diseases, the identification of emerging risks in occupational health, the identification and description of occupational risk situations, and helps to define priority actions [13,14,15,16].

Between 2001 and 2012, the RNV3P logged 294,243 consultations. Due to the size and importance of the RNV3P database, encoding data in a consistent manner is essential, especially for statistical analyses. The aim of using a standardized and shared terminology is to describe and characterize work situations related to occupational diseases, to develop prevention strategies for occupational risks, and to facilitate the exchange and sharing of information between different stakeholders. Thus, a common classification of occupational exposure factors was developed which is freely available for all healthcare professionals, including physicians and public health specialists [14]. In 2012, the most frequently encountered health problems in occupational consultation centers were related to mental and behavioral disorders (19%). However, no classification of occupational exposure factors for mental and behavioral disorders at work is currently available. Knowledge of the impact of the psychosocial work environment on mental and behavioral disorders can assist occupational physicians in the identification and description of occupational risk situations, and helps to define priority actions.

The primary aim was to build a thesaurus of “Organizational, Relational, Ethical and other Contributing Factors” (FOREC) that contribute to the onset of mental and behavioral disorders. In this article we present the methodology that led to its creation. The secondary aim was to describe the results obtained after using the FOREC thesaurus in clinical practice during consultations for work-related mental disorders.

## Methods

### Development of the FOREC thesaurus

The French agency for Food, Environmental and Occupational Health and Safety (ANSES) initiated and supervised a multidisciplinary working group to build the FOREC thesaurus. This working group was set up with the representatives of the main occupational and public health actors, such as the ANSES, the French National Health Insurance System (CNAM), Occupational Disease Centers, Occupational Health Services, the French Institute for Public Health Surveillance (InVS), the Interdepartmental Center of Health and Occupational Medicine in Factories (CISME), and the French Institute for Research and Security (INRS). This work was collaborative. The representatives of the main occupational and public health actors who attended the meetings for the creation of the FOREC thesaurus were nationally recognized experts in health, occupational medicine, mental disorders, and work-related stressors (organization, relation, or ethics). Specialized physicians from Occupational Diseases Centers of university hospitals, who undertake mental health consultations in the workplace [17,18], participated in the meetings. For two years, they were asked to list all organizational, relational, ethical and other contributing occupational factors linked with mental and behavioral disorders that were identified during their mental health consultations. Other actors also proposed other putative contributing factors from their own personal experiences. Eleven national meetings were held over three years. Firstly, the occupational exposure factors that could promote mental health and prevent mental and behavioral disorders were listed. All decisions regarding the inclusion of factors within the FOREC thesaurus were approved on a consensus basis. They were then classified, which led to an updated FOREC thesaurus.

### Presentation of the FOREC thesaurus

The classification was proposed by members of the multidisciplinary working group on a consensus basis. Occupational factors linked with mental and behavioral disorders were grouped into clinically relevant headings and sections based on ICD-10. The multidisciplinary working group proposed a FOREC thesaurus composed of six chapters: inherent demand of the work, functional organization of the business, relations at work and violence, personal ethics, ethics of the business, and other contributing factors. The first five chapters classify the professional situation inside the enterprise. The sixth chapter concerns the “contributing factors” that are related to the person’s status or are totally external to the enterprise. These six chapters are divided into a total of 34 subchapters. Subchapters are coded with 3 digits and are divided into 186 items in total. Items are coded with 4, 5 or 6 digits. The FOREC thesaurus is presented in Table 1 in its entirety.

**Table 1:** Organizational, relational, ethical and other contributing factors–Thesaurus completed to 4, 5 or 6 digits.

Code				Heading
**70**				**Inherent demand of the work**
***700***				***Work schedule***
	7000			Shift work *(2x8*, *3x8*, *5x8* …*)*
	7001			Night work
		70010		Regular night work *(>5 nights per month)*
		70011		Occasional night work
	7002			On-call working
	7003			Working on Sundays and public holidays
	7004			Length of working day consistently in excess of 10 hours
	7005			Split shifts *(divisible or split working day)*
	7006			Weekly rest period regularly less than 48 hours
	7007			Unpredictability of working schedule
	7009			Other working schedule capable of causing disturbance to health
***701***				***Business travel***
	7010			Business travel *(mission)* disturbing social life without sleeping out
	7011			Business travel *(mission)* disturbing social life with sleeping out
	7012			Business travel *(mission)* disturbing chronobiology *(jet lag)*
	7019			Other business travel capable of causing disturbance excluding chosen journeys/commuting cited in 7533.
***702***				***Other specific features imposed by the work***
	7020			On call by telephone or email
		70200		On call by telephone *(or SMS or email)* only
		70201		On call by telephone *(or SMS or email)* with call out
		70209		Other on call
	7021			Involuntary part-time work
	7022			Imposed teleworking
	7023			Imposed working from home
	7024			Imposed temporary work
	7025			Imposed overtime
	7026			Requests at any time outside of working hours
		70260		Requests at any time outside of working hours by clients
		70261		Requests at any time outside of working hours by management or colleagues
	7029			Other specific imposed conditions capable of causing disturbance
***703***				***Distinctive feature of the work***
	7030			Poor quality of work content
		70300		Monotonous work, little or no creativity
		70301		Versatility of tasks resulting in a lack of identity *(stand in)*
		70309		Other features of poor quality of work content
	7031			Specific demand of work content
		70310		Activities requiring alertness, concentration, very close attention
		70311		Function involving high human, financial or safety responsibilities *(increase in responsibilities)*
		70312		Regular contact with the public
		70313		Working alone *(work with a lack of contact causing feelings of isolation)*
		70314		Variability, unpredictability of workload
		70315		Work with strong emotional loading *(e*.*g*. *empathy*, *contact with people who are suffering)*
		70316		Work requiring continuous or excessive control of emotions *(facticity*, *inauthenticity*, *hiding emotions)*
		70317		Working under imposed time constraints *(assembly line work*, *high throughput*, *performance based wages*, *pace imposed)*
		70318		Fragmented or segmented work: multiple, concurrent tasks or frequent interruptions.
		70319		Other demands of work content capable of causing disturbance
	7039			Other distinctive feature of the work capable of causing disturbance
***709***				***Other general inherent demand of the work capable of causing disturbance***
**71**				**Functional organization of the business**
***710***				***Change in the organization and specific approach of management***
	7100			Major restructuring in preceding or coming months
		71000		Elimination of position
		71001		Outsourcing of business
		71002		Takeover
	7101			Change of personnel
		71010		Change of colleagues
		71011		Change of management
		71019		Other change of personnel
	7102			Change of methods
		71020		Change of management methods
		71021		Change of production methods
	7103			Non-regulated matrix management or cross-cutting project
	7109			Other change in the organization capable of causing disturbance
***711***				***Insufficient or excessive workload experienced***
	7110			Excessive workload experienced
		71100		Excessive workload experienced during working hours
		71101		Excessive workload experienced requiring working at home
	7111			Insufficient workload experienced
	7119			Other workload conditions capable of causing disturbance
***712***				***Excessive procedures and supervision***
	7120			Procedures perceived as excessive
	7121			Supervision perceived as excessive
	7122			Continuous supervision by material means *(video surveillance*, *computer*, *recording*, *informer)*
	7129			Other procedure or supervision perceived as excessive
***713***				***Low decision latitude in the organization of their work***
***714***				***Few opportunities to learn or develop their skills***
***715***				***Lack of recognition (encouragement*, *congratulations etc*.*) or reward (e*.*g*. *salary*, *promotion*, *annual appraisal)***
	7150			Perceived deficiencies in verbal expression, lack of expression of recognition in oral or written form
	7151			Perceived deficiency of salary
	7152			Perceived deficiency *(lack or delay)* of promotion
	7153			Perceived deficiency of recognition of title or of degree
	7159			Other perceived deficiency of recognition or of reward
***716***				***Insufficiency of resources***
	7160			Mismatch objective/resources
	7161			Insufficient training in connection with the task to be undertaken
	7162			Failings in communications flow
	7163			Objective deficiency of management *(lack of management personnel or overly distant management)*
	7164			Objective deficiency of non-managerial personnel, of work colleagues *(unfilled position*, *absence not covered)*
	7165			Slippage of task and responsibility (ambiguity of roles)
	7169			Other insufficiencies of resources
***717***				***Dysfunctions in the instructions of management***
	7170			Content of the work poorly defined *(absence of job description or procedures)*
	7171			Paradoxical instructions
	7172			Regularly exceeding contractual hours, unpaid, unrecovered overtime
	7173			Perceived ambiguous positioning of management
	7174			Management perceived as evasive *(failure to arbitrate*, *not taking decisions*, *etc)*
	7175			Objectives seen as unattainable *(pressure*, *not objective)*
	7179			Other dysfunctions in the instructions from management capable of causing disturbance
***718***				***Transfer to another position or another site (or announced in the 3 months preceding the first signs)***
	7180			Transfer for a determined period
		71800		Transfer for a determined period not requiring family relocation
		71801		Transfer for a determined period requiring family relocation
	7181			Transfer for an undetermined period
		71810		Transfer for an undetermined period not requiring family relocation
		71811		Transfer for an undetermined period requiring family relocation
	7182			Imposed redeployment
		71820		Imposed redeployment to another position at the same site
		71821		Imposed redeployment to another position at another site
	7189			Other transfer to another position or another site
***719***				***Other general features of the functional organization of the business capable of causing disturbance***
**72**				**Relations at work and violence**
***720***				***Quality of relations at work***
	7200			Deleterious relationships experienced
		72000		Deleterious relationship with management experienced
			720001	Deleterious relationship with management with constant criticism experienced
			720002	Deleterious relationship with management through lack of being heard experienced
			720003	Deleterious relationship with management through asymmetric communications experienced
			720004	Deleterious relationship with management with implicit threat of dismissal experienced
		72001		Deleterious relationship with the work group or peers experienced *(sidelined*, *categorical divide)*
		72002		Deleterious relationship in isolation with a colleague experienced
		72003		Deleterious relationships experienced after undergoing disciplinary measures *(suspension* …*)*
	7201			Deficiency of support experienced
		72010		Deficiency of support from management experienced
		72011		Deficiency of support from the work group or peers experienced
	7209			Other qualitative feature of relations capable of causing disturbance
***721***				***External violence (persons outside of the workplace)***
	7210			Verbal aggression (*external violence*)
		72010		Verbal aggression without credible threat of death (*external violence*)
		72011		Verbal aggression with credible threat of death (*external violence*)
	7211			Physical aggression
	7212			Robbery, hold-up
	7213			Witnessing a traumatic event
	7219			Other external violence
***722***				***Internal violence (another company employee)***
	7220			Verbal aggression (*internal violence)*
		72200		Verbal aggression without credible threat of death (*internal violence)*
		72201		Verbal aggression with credible threat of death (*internal violence)*
	7221			False accusation experienced
		72210		False accusation experienced without a procedure
		72211		False accusation experienced with involvement in a procedure
	7222			Aggression, physical violence suffered
	7223			Traumatic event experienced as a witness or through an account received
		72230		Witnessing verbal or physical aggression
		72231		Witnessing workplace death, excluding suicide
		72232		Witnessing a suicide
			722320	Witnessing a successful suicide at work
			722321	Witnessing a suicide attempt at work
		72233		Received account of verbal or physical aggression or of a suicide attempt
			722330	Received account of verbal or physical aggression or of a suicide attempt linked to work, outside of workplaces (*threats during journeys*)
			722331	Received account of verbal OR physical aggression or of a suicide attempt occurring in the workplace
		72234		Received account of a successful suicide
			722340	Received account of a successful suicide linked to work outside of the workplace
			722341	Received account of a successful suicide in the workplace
		72235		Received account of a death *(excluding suicide)* linked to work *(colleagues)* wherever the place of occurrence
		72239		Other traumatic event related to work
	7224			Sexual harassment experienced
	7225			Discrimination experienced *(gender*, *age*, *sexual orientation*, *etc*.*)*
	7226			Bullying at work experienced
	7227			Deskilling
	7228			Sidelined
	7229			Other internal violence
***729***				***Other general features of relations at work capable of causing disturbance***
**73**				**Personal ethics–conflict of values**
***730***				***Performing an act going against their principles (miss-selling*, *making redundancies)***
***731***				***Being a powerless witness to acts going against their principles***
***732***				***Lacking resources or time to do quality work***
***739***				***Other conflict of values relating to personal ethics***
**74**				**Ethics of the business**
***740***				***General level of safety or a low safety culture***
***741***				***General level of hygiene or poor hygiene culture***
***742***				***Lack of means***
	7420			Lack of collective means of protection
	7421			Lack of individual means of protection
***743***				***Lack of respect in verbal communications***
***749***				***Other ethical failing of the business capable of causing disturbance***
**75**				**Other contributing factors**
***750***				***Particular medical or social status capable of altering relationships***
	7500			Inadequate or inappropriate consideration of limitations of ability *(excluding disability)*
	7501			Limitation of ability not accepted by the employee
	7502			Return after absence
		75020		Return after a break in working due to illness
		75021		Return after an accident at work/occupation related illness/disease
		75022		Return after maternity leave
		75023		Return after parental leave
		75024		Return after annual leave
		75025		Return after individual training leave
	7503			Person recognized as having a disability
	7509			Other medical or social status able to alter relations
***751***				***Claims for entitlements including*: *claims concerning leave*, *training*, *bonuses not being received*, *payment of overtime*, *signing a petition***
	7510			Action taken as a result of social commitment or elective mandate
	7511			Action taken in a personal capacity
	7519			Other feature of claiming rights
***752***				***Taking a personal stance or action challenging the company***
	7520			Denouncing supposed or alleged dishonest actions connected with professional activities
	7521			Externalization of an internal company issue *(with a labor inspectorate*, *a lawyer*, *etc)*
	7529			Other personal stance or action challenging the company
***753***				***Specific chosen working conditions***
	7530			Multiple employers
	7531			Chosen teleworking
	7532			Chosen working at home
	7533			Home—work commute
		75330		Home—work commute >2 hours per day
		75331		Home—work commute >3 hours per day
	7534			Overqualified at work
	7535			Desired redeployment to another position
		75350		Desired redeployment to another position at the same site
		75351		Desired redeployment to another position at another site
	7536			Position not meeting aspirations but accepted for economic reasons
	7537			Chosen part-time work
	7538			Chosen temporary work
	7539			Other specific chosen working condition able to cause disturbance
***754***				***Contributing factor linked to the business (social context*, *economic context)***
	7540			Unfavorable socio-economic context
		75400		Unfavorable social context *(social upheaval*, *strike*, *periods of notice)*
		75401		Unfavorable economic context *(temporary lay-offs*, *wage freezes*, *company financial difficulties)*
		75409		Other unfavorable socio-economic context
	7541			Conventional procedure of contract termination or leaving voluntarily in progress (only code if unfavorably experienced)
	7542			Job insecurity
	7543			Involvement in a termination procedure
		75430		Involvement in a redundancy procedure
		75431		Involvement in an individual termination procedure
	7544			Family business context or of specific links between the individual and management
	7549			Other unfavorable context linked to the company
***759***				***Other general feature constituting a contributing factor***

The first chapter, *“Inherent demand of the work”*, concerns all professional constraints that cannot be dissociated from the activity, but that may be the cause of various disorders. For example, being on call in a hospital can disturb social life or cause insomnia. This chapter is divided into 5 subchapters and consists of 44 items in total.

The second chapter, called *“Functional organization of the business”*, also concerns strong professional constraints. It classifies occupational exposure factors, such as organizational changes, restructuring, insufficient or excessive workload, and workplace transfers. This chapter is divided into 10 subchapters and consists of 51 items in total.

The third chapter is entitled *“Relations at work and violence”* (referring to instances such as deleterious relationships experienced with management, verbal aggressions, and sidelining). In this chapter, the notions of individual experiences of a more subjective nature are presented. This chapter is divided into 4 subchapters and consists of 47 items in total.

Chapters four and five are respectively related to *“Personal ethics”* (e.g. performing an act going against one’s principles) and *“Ethics of the business”* (e.g. lack of respect in verbal communications). These chapters provide justification of ethical issues based on case studies.

The sixth chapter, *“Other contributing factors”*, regroups factors that may be viewed as directly related to a person (such as a return after parental leave) or totally external to the enterprise (such as an unfavorable socio-economic context). This chapter is divided into 6 subchapters and consists of 42 items in total.

### Multicenter study

To check the exhaustiveness and clinical relevance of the FOREC thesaurus, we aimed to describe organizational, relational, ethical and other contributing occupational factors linked with mental and behavioral disorders identified during consultations for mental health issues at work. A French multicenter study (Clermont-Ferrand, Créteil, Toulouse, Bordeaux, Garches) was therefore implemented. The Occupational Disease Centers at these University Hospitals were chosen because they shared similar characteristics relating to the management of mental health consultations in the workplace. Included workers were addressed to those consultations by their general practitioner or their occupational physician because of a presumed diagnosis of mental health issues at work [17,18]. They were included over a period of twelve consecutive months. There were no exclusion criteria and all patients who attended a consultation for mental health issues at work were included. The protocol was approved by the ethics committee of the University Hospital of Clermont-Ferrand (approval number: n° 2015CE/69). Socio-demographic, occupational and clinical data were retrieved during two interviews, one with a psychologist and a nurse and a second with an occupational physician and a psychiatrist. The final diagnosis of a mental and behavioral disorder was made during this second, specialized, consultation for mental health issues at work, which also established a link between the issues and professional activity. The link was based on the specialist’s judgment, during the medical examination. Mental and behavioral disorder were coded according to the International Classification of Diseases 10th Revision (ICD-10) and all physicians were trained in this coding by the same organization (ANSES). A detailed medical report was systematically written by the occupational physicians and the psychiatrists for each consultation. The medical report mentioned the diagnosis and the socio-demographic, occupational and clinical data, as well as occupational exposure factors linked with the mental health issues of the workers. The medical reports identifiers were coded prior to analysis. One author (CL) reviewed all coded medical reports from all centers. The author identified and retrospectively coded the data and factors contributing to the workers’ health-related issues using the FOREC thesaurus from the perception of the worker.

### Statistical analysis

Statistical analyses were performed using Stata software, version 13 (StataCorp, College Station, TX, US). Continuous data were expressed as mean ± standard deviation and categorical parameters as frequencies (associated percentages).

## Results

We recruited 323 workers (31.3% males) with an average age of 44.9±9.2. The most commonly encountered diagnoses in the consultations for mental health issues at work were ‘generalized anxiety’ (106 workers, 32.8%) and ‘moderate depressive episodes’ (86 workers, 26.7%) (Table 2).

**Table 2:** Characteristics of the workers.

		n = 323
**Demographic characteristics**		
	Gender (male)	101 (31.3)
	Age (years)	44.9 ± 9.2
**Disorders encountered**		
	F320 –Mild depressive episode	9 (2.8)
	F321 –Moderate depressive episode	86 (26.7)
	F322 –Severe depressive episode without psychotic symptoms	39 (12.1)
	F410 –Panic disorder	3 (0.9)
	F411 –Generalized anxiety	106 (32.8)
	F412 –Mixed anxiety and depressive disorder	47 (14.6)
	F419 –Unspecified anxiety disorder	13 (4.0)
	F431 –Post-traumatic stress disorder	2 (0.6)
	Z730 –Burn-out	11 (3.4)
	Z03 –No pathology	3 (0.9)
	Other pathology	2 (0.6)
	No answer	2 (0.6)

Data are presented as frequencies (associated percentages) or as mean ± standard deviation.

Encountered diseases are presented with their ICD-10 corresponding code.

Other pathology: lateral epicondylitis (M77 1) and irritability and anger (R454).

During the study period, 1357 occupational factors linked with mental and behavioral disorders were identified, i.e. an average of 4.2 factors per worker. Among them, 575 (42.4%) were relational, 515 (37.9%) were organizational and the remaining 12.2% represented other contributing factors (Table 3). All identified factors were successfully encoded using the thesaurus. Two subchapters were not found in medical reports: “Other general inherent demands of the work capable of causing disturbance” (subchapter 709) and “General level of hygiene or poor hygiene culture” (subchapter 741).

**Table 3:** Number of factors identified for each chapter and subchapter.

		n = 1357
**70**	**Inherent demand of the work**	**27 (2.0)**
700	Work schedule	8 (0.6)
701	Business travel	2 (0.1)
702	Other specific features imposed by the work	2 (0.1)
703	Distinctive feature of the work	15 (1.1)
709	Other general inherent demand of the work capable of causing disturbance	0 (0.0)
**71**	**Functional organization of the business**	**515 (37.9)**
710	Change in the organization and specific approach of management	148 (10.9)
711	Insufficient or excessive workload experienced	67 (4.9)
712	Excessive procedures and supervision	22 (1.6)
713	Low decision latitude in the organization of their work	17 (1.3)
714	Few opportunities to learn or develop their skills	4 (0.3)
715	Lack of recognition (encouragement, congratulations etc.) or reward (e.g. salary, promotion, annual appraisal)	101 (7.5)
716	Insufficiency of resources	66 (4.9)
717	Dysfunctions in the instructions of management	61 (4.5)
718	Transfer to another position or another site (or announced in the 3 months preceding the first signs)	25 (1.9)
719	Other general features of the functional organization of the business capable of causing disturbance	4 (0.3)
**72**	**Relations at work and violence**	**575 (42.4)**
720	Quality of relations at work	306 (22.6)
721	External violence (persons outside of the workplace)	17 (1.3)
722	Internal violence (another company employee)	250 (18.4)
729	Other general features of relations at work capable of causing disturbance	2 (0.1)
**73**	**Personal ethics–conflict of values**	**57 (4.2)**
730	Performing an act going against their principles (miss-selling, making redundancies)	12 (0.9)
731	Being a powerless witness to acts going against their principles	19 (1.4)
732	Lacking resources or time to do quality work	11 (0.8)
739	Other conflict of values relating to personal ethics	15 (1.1)
**74**	**Ethics of the business**	**17 (1.3)**
740	General level of safety or a low safety culture	8 (0.6)
741	General level of hygiene or poor hygiene culture	0 (0.0)
742	Lack of means	4 (0.3)
743	Lack of respect in verbal communications	3 (0.2)
749	Other ethical failing of the business capable of causing disturbance	2 (0.1)
**75**	**Other contributing factors**	**166 (12.2)**
750	Particular medical or social status capable of altering relationships	45 (3.3)
751	Claims for entitlements including: claims concerning leave, training, bonuses not being received, payment of overtime, signing a petition	11 (0.8)
752	Taking a personal stance or action challenging the company	73 (5.4)
753	Specific chosen working conditions	3 (0.2)
754	Contributing factor linked to the business (social context, economic context)	32 (2.4)
759	Other general feature constituting a contributing factor	2 (0.1)

Data are presented as frequencies (associated percentages).

Relational factors were the most common in cases of depressive episodes (41.7%) and anxiety disorders (44.4%), followed by managerial factors (Table 4). Managerial factors were most frequently identified in cases of post-traumatic stress disorder (62.5%) and burn-out (59.6%).

**Table 4:** Number of factors identified for each chapter, according to work-related challenges.

		Depressive episode	Anxiety disorders	PTSD	Burn-out	No pathology	Other pathology	No answer
		(n = 575)	(n = 699)	(n = 8)	(n = 47)	(n = 11)	(n = 5)	(n = 12)
70	Inherent demand of the work	10 (1.8)	12 (1.7)	0 (0.0)	5 (10.6)	0 (0.0)	0 (0.0)	0 (0.0)
71	Functional organization of the business	227 (39.5)	248 (35.5)	5 (62.5)	28 (59.6)	2 (18.2)	0 (0.0)	5 (41.7)
72	Relations at work and violence	240 (41.7)	310 (44.4)	2 (25.0)	10 (21.3)	6 (54.5)	2 (40.0)	5 (41.7)
73	Personal ethics–conflict of values	22 (3.8)	33 (4.7)	0 (0.0)	2 (4.3)	0 (0.0)	0 (0.0)	0 (0.0)
74	Ethics of the business	7 (1.2)	8 (1.1)	1 (12.5)	1 (2.1)	0 (0.0)	0 (0.0)	0 (0.0)
75	Other contributing factors	69 (12.0)	88 (12.6)	0 (0.0)	1 (2.1)	3 (27.3)	3 (60.0)	2 (16.6)

Data are presented as frequencies and associated percentages.

ICD-10 corresponding codes were F320, F321, F322 for depressive episode; F410, F411, F412, F419 for anxiety disorders; F431 for post-traumatic stress disorder (PTSD); Z730 for burn-out; Z03 for no pathology; M771 (lateral epicondylitis) and R454 (irritability and anger) for other pathologies.

## Discussion

This study presents the first thesaurus of occupational exposure factors responsible for mental health issues at work, grouped into clinically relevant headings and sections.

### Consequences to mental health following exposure to occupational factors

Mental health issues at work are a public health concern. More interestingly, some factors related to work, such as organizational, relational, ethical and other contributing factors were directly linked with some health-related issues. For example, it has been shown that cardiovascular diseases were associated with job strain [19], low decision latitude [20], low reward [21] and low social support [22,23]. It has also been reported that low social support positively predicts depression [24]. Similarly, changes in organization (10.9% of the 1357 identified factors) and conflict of loyalties resulting from workplace changes may also lead to suicide [25]. Moreover, it has long been recognized that depression and anxiety share similarities in their pathogenesis [26]. Among the mental disorders identified in the present study, anxiety and depression seem to share the same exposure profile, i.e. relational (~40%) [27], whereas burn-out was mainly linked with organizational factors (~60%), as previously reported [28,29,30].

### Occupational factors linked with mental and behavioral disorders

In our study, identified factors were mainly relational (42.4%). Relational regulation theory has previously been proposed as a novel way to improve mental and behavioral disorders [31]. On the other hand, inherent work demands were infrequently cited (2%), which consequently may not be a major source of mental health issues at work. In our study, the inherent work demands that cannot be dissociated from the professional activity seem to be accepted and well tolerated by workers, which might appear contradictory to some previous literature [20]. We demonstrated that 1.3% of the factors identified were related to low decision latitude, 7.5% to lack of recognition or reward and 4.9% to perceptions of a deficiency of support. Supervisor support has previously been shown to buffer the impact of excessive work demands [31]. One hundred and sixty-six contributing and contextual factors (12.2%) showed some new insights into particular medical or social status (such as perceived limitations of ability, return after absence) that may be capable of altering relationships or altering work-related self-efficacy. Studying those factors could provide possibilities for changing practices in the workplace.

### Recommendation

The FOREC thesaurus has a worldwide application as the use of a standardized and shared terminology is needed to describe at-risk occupational factors that generate mental and behavioral disorders. Identifying those factors is essential for effective prevention in the workplace and must be based on useful evidence-based information in order to help to define priority actions. At-risk workers should receive follow-ups from occupational physician [32,33], and may benefit from a targeted intervention on the occupational factors that have been identified as generating mental and behavioral disorders [34].

### Limitations

There are limitations to this study. Some subchapters of the thesaurus may seem less relevant because they were less common. However, a detailed thesaurus is required for exhaustively encoding exposures. This thesaurus can be improved, as are other thesauruses which are continuously enriched by the French National Occupational Diseases Monitoring and Prevention Network (RNV3P) [35,36,37], which also guarantees that they will remain current. The sample size used for describing results from the FOREC thesaurus may seem low, however workers were recruited at the Occupational Diseases Centers of the University Hospitals’ during specialized consultations for mental health issues at work [17,18]. Moreover, a relevant number of occupational factors linked with mental and behavioral disorders were identified (over one thousand), providing substantial data. Variability between independent coders also needs to be assessed. Comparing encoding between a physician and an administrative employee would be of interest. Furthermore, personality traits of the coder may influence data encoding and should be evaluated. Further studies are needed to assess the profile of occupational exposure factors with regard to socio-professional occupations and workers’ demographic characteristics.

## Conclusion

We built the first thesaurus of “Organizational, Relational, Ethical and other Contributing Factors” (FOREC) that may help to generate profiles of mental and behavioral disorders at work. Using the FOREC thesaurus in clinical practice during consultations for work-related mental health issues has shown that the factors identified during previous consultations were successfully encoded (all factors were described in the thesaurus). The FOREC thesaurus may provide a worldwide standardized and shared terminology, which will help to understand the impact of the psychosocial work environment on the onset of mental and behavioral disorders related to work. The identification and description of occupational risk situations can assist occupational physicians in defining priority actions in the workplace to address mental health issues.

## Supporting information

S1 Appendix French version of the FOREC thesaurus. (DOCX)

S1 Database Titles of columns are written without abbreviations. (XLSX)

S1 Table Number of workers per occupational group and per level of function according the ISCO-08 classification (n = 322 because of one missing value). (DOCX)
