# Supplementary material for: Occupational exposure factors for mental and behavioral disorders at work: The FOREC thesaurus
Source: PLoS One. 2018 Jun 21;13(6):e0198719. doi: 10.1371/journal.pone.0198719 (PMC6013225; doi:10.1371/journal.pone.0198719)
Supplement: S1 Appendix — (DOCX) [file pone.0198719.s001.docx]

**Appendix 1.** French version of the FOREC Thesaurus

Impact de l’environnement psychosocial de travail sur les troubles mentaux et du comportement : **Facteurs Organisationnels, Relationnels, Ethiques et autres facteurs Contributeurs – Thésaurus FOREC complet à 4, 5 ou 6 digits**

| **Code** | | | | **Intitulé** |
| --- | --- | --- | --- | --- |
| **70** | | | | **Exigences inhérentes à l'activité** |
| ***700*** | | | | ***Horaires de travail*** |
|  | 7000 | | | Travail posté (2x8, 3x8, 5x8...) |
|  | 7001 | | | Night work |
|  |  | 70010 | | Travail de nuit régulier (> 5 nuits par mois) |
|  |  | 70011 | | Travail de nuit occasionnel |
|  | 7002 | | | Travail de garde |
|  | 7003 | | | Travail dimanche et jours fériés |
|  | 7004 | | | Amplitude horaire de travail continu supérieure à l0h |
|  | 7005 | | | Travail fractionné (sécabilité ou travail en coupure) |
|  | 7006 | | | Repos hebdomadaire régulièrement inférieur à 48h |
|  | 7007 | | | Imprévisibilité des horaires de travail. |
|  | 7009 | | | Autres horaires de travail pouvant générer une nuisance sur la santé |
| ***701*** | | | | ***Déplacements professionnels*** |
|  | 7010 | | | Déplacements professionnels (missions) perturbant la vie sociale sans découcher |
|  | 7011 | | | Déplacements professionnels (missions) perturbant la vie sociale avec découcher |
|  | 7012 | | | Déplacements professionnels (missions) perturbant la chronobiologie (décalage horaire) |
|  | 7019 | | | Autres déplacements professionnels générant une nuisance à l'exclusion des trajets choisis cités en 7533 |
| ***702*** | | | | ***Autres modalités particulières imposées de travail*** |
|  | 7020 | | | Astreintes par téléphone ou email |
|  |  | 70200 | | Astreintes par téléphone *(ou SMS ou email)* seulement |
|  |  | 70201 | | Astreintes par téléphone *(ou SMS ou email)* avec intervention |
|  |  | 70209 | | Autres astreintes |
|  | 7021 | | | Travail à temps partiel imposé |
|  | 7022 | | | Télétravail imposé |
|  | 7023 | | | Travail à domicile du salarié imposé |
|  | 7024 | | | Travail temporaire imposé |
|  | 7025 | | | Travail à temps partiel imposé |
|  | 7026 | | | Heures supplémentaires imposées à n’importe quel moment |
|  |  | 70260 | | Heures supplémentaires imposées à n’importe quel moment par les clients |
|  |  | 70261 | | Heures supplémentaires imposées à n’importe quel moment par la hiérarchie ou les collègues |
|  | 7029 | | | Autres modalités particulières imposées pouvant générer une nuisance |
| ***703*** | | | | ***Caractéristiques propres à l'activité*** |
|  | 7030 | | | Pauvreté de contenu du travail |
|  |  | 70300 | | Travail monotone, peu ou pas créatif |
|  |  | 70301 | | Polyvalence des tâches entraînant un déficit d'identité (bouche trou) |
|  |  | 70309 | | Autres caractéristiques de pauvreté du contenu du travail |
|  | 7031 | | | Exigence particulière de contenu |
|  |  | 70310 | | Activités demandant une vigilance, une concentration, une attention soutenue |
|  |  | 70311 | | Fonctions à forte responsabilité assumée humaine, financière ou de sécurité (*et* *augmentation des responsabilités*) |
|  |  | 70312 | | Contact régulier avec le public |
|  |  | 70313 | | Travail isolé (*travail avec manque de contact pouvant générer un sentiment d'isolement*) |
|  |  | 70314 | | Variabilité, imprévisibilité de la charge de travail |
|  |  | 70315 | | Travail avec forte charge émotionnelle (*ex : empathie, contact avec personne* *en souffrance*) |
|  |  | 70316 | | Travail imposant un contrôle permanent ou excessif des émotions (*facticité, inauthenticité cacher ses émotions*) |
|  |  | 70317 | | Travail sous contraintes de temps imposées (*travail à la chaîne, cadences élevées, salaires au rendement, rythme imposé*) |
|  |  | 70318 | | Travail dit "haché" ou segmenté : Tâches multiples, concomitantes ou interruptions fréquentes |
|  |  | 70319 | | Autres exigences de contenu pouvant générer une nuisance |
|  | 7039 | | | Other distinctive feature of the work capable of causing disturbance |
| ***709*** | | | | ***Autres exigences générales inhérentes à l'activité pouvant générer une nuisance*** |
| **71** | | | | **Organisation fonctionnelle de l'activité (management)** |
| ***710*** | | | | **Changements dans l'organisation et modalités particulières de management** |
|  | 7100 | | | Restructuration importante dans les mois écoulés ou à venir |
|  |  | 71000 | | Suppression de postes |
|  |  | 71001 | | Externalisation des processus |
|  |  | 71002 | | Rachat |
|  | 7101 | | | Changement des personnes |
|  |  | 71010 | | Changement de collègue |
|  |  | 71011 | | Changement de hiérarchie |
|  |  | 71019 | | Autres changements des personnes |
|  | 7102 | | | Changement de méthode |
|  |  | 71020 | | Changement de méthode de management |
|  |  | 71021 | | Changement de méthode de production |
|  | 7103 | | | Management matriciel non régulé ou projet transversal |
|  | 7109 | | | Autres changements dans l'organisation pouvant générer une nuisance |
| ***711*** | | | | ***Surcharge ou sous-charge de travail ressentie*** |
|  | 7110 | | | Surcharge de travail ressentie |
|  |  | 71100 | | Surcharge de travail ressentie durant les heures de travail |
|  |  | 71101 | | Surcharge de travail ressentie nécessitant de travailler à la maison |
|  | 7111 | | | Sous-charge de travail ressentie |
|  | 7119 | | | Autres modalités de charge de travail pouvant générer une nuisance |
| ***712*** | | | | ***Procédures et contrôles excessifs*** |
|  | 7120 | | | Procédures perçues comme excessives |
|  | 7121 | | | Contrôles perçus comme excessifs |
|  | 7122 | | | Contrôles continus par des moyens matériels (vidéosurveillance, informatiques, enregistrements, mouchard...) |
|  | 7129 | | | Autres procédures ou contrôles perçus comme excessifs |
| ***713*** | | | | ***Faible latitude de décision dans l'organisation de son travail*** |
| ***714*** | | | | ***Peu de possibilités d'apprendre ou de développer ses compétences*** |
| ***715*** | | | | ***Déficit de reconnaissance (encouragements, félicitations...) ou de récompense (ex: salaires, promotion, entretien annuel d'évaluation)*** |
|  | 7150 | | | Déficit perçu d'expression verbale, manque d'expression de reconnaissance sous forme orale ou écrite |
|  | 7151 | | | Déficit perçu de salaire |
|  | 7152 | | | Déficit perçu (*manque ou retard*) de promotion professionnelle |
|  | 7153 | | | Déficit perçu de reconnaissance de titre ou de diplôme |
|  | 7159 | | | Autres déficits perçus de reconnaissance ou de récompense |
| ***716*** | | | | ***Insuffisance de moyens*** |
|  | 7160 | | | Inadéquation objectifs/moyens |
|  | 7161 | | | Insuffisance de formation en rapport avec les tâches à exercer |
|  | 7162 | | | Défaut de circulation de la communication |
|  | 7163 | | | Déficit objectif d'encadrement (*il manque du personnel d'encadrement ou encadrement trop éloigné*) |
|  | 7164 | | | Déficit objectif du personnel non cadre, des collègues de travail (*poste non pourvu, absence non remplacée*) |
|  | 7165 | | | Glissements des tâches et des responsabilités (*ambigüité des rôles*) |
|  | 7169 | | | Autres insuffisances de moyens |
| ***717*** | | | | ***Dysfonctionnement des prescriptions de la hiérarchie*** |
|  | 7170 | | | Contenu du travail objectivement mal défini (*absence de fiche de poste ou de procédures*) |
|  | 7171 | | | Injonctions paradoxales |
|  | 7172 | | | Dépassement habituel de l'horaire contractuel, heures supplémentaires non payées, non récupérées |
|  | 7173 | | | Positionnement de la hiérarchie perçu comme ambigu |
|  | 7174 | | | Hiérarchie perçue comme fuyante (*défaut d'arbitrage, pas de prise de décision...*) |
|  | 7175 | | | Autres dysfonctionnements des prescriptions de la hiérarchie pouvant générer une nuisance |
|  | 7179 | | | Contenu du travail objectivement mal défini (*absence de fiche de poste ou de procédures*) |
| ***718*** | | | | ***Mutation sur autre poste ou autre site (ou annonce faite dans les 3 mois précédent les premiers signes)*** |
|  | 7180 | | | Mutation pour une durée déterminée |
|  |  | 71800 | | Mutation pour une durée déterminée n'imposant pas le déménagement de la famille |
|  |  | 71801 | | Mutation pour une durée déterminée imposant le déménagement de la famille |
|  | 7181 | | | Mutation pour une durée indéterminée |
|  |  | 71810 | | Mutation pour une durée indéterminée n'imposant pas le déménagement de la famille |
|  |  | 71811 | | Mutation pour une durée indéterminée imposant le déménagement de la famille |
|  | 7182 | | | Réaffectation imposée sur un autre poste |
|  |  | 71820 | | Réaffectation imposée sur un autre poste du même site |
|  |  | 71821 | | Réaffectation imposée sur un autre poste d’un autre site |
|  | 7189 | | | Autres mutations sur un autre site ou sur un autre poste |
| ***719*** | | | | ***Autres modalités générales d’organisation fonctionnelle de l'activité pouvant générer une nuisance*** |
| **72** | | | | **Relations au travail et violence** |
| ***720*** | | | | ***Qualité des relations au travail*** |
|  | 7200 | | | Relations vécues délétères |
|  |  | 72000 | | Relations vécues délétères avec la hiérarchie |
|  |  |  | 720001 | Relations vécues délétères avec la hiérarchie avec des critiques permanentes |
|  |  |  | 720002 | Relations vécues délétères avec la hiérarchie avec un manque d'écoute |
|  |  |  | 720003 | Relations vécues délétères avec la hiérarchie avec une asymétrie de communication |
|  |  |  | 720004 | Relations vécues délétères avec la hiérarchie avec menace implicite de licenciement |
|  |  | 72001 | | Relation vécue délétère dans le collectif de travail ou avec les pairs *(ex : mise à l'écart, clivage catégoriel)* |
|  |  | 72002 | | Relation vécue délétère avec un collègue de façon isolée |
|  |  | 72003 | | Relation vécue délétère après des mesures disciplinaires *(suspension...)* |
|  | 7201 | | | Déficit vécu de soutien |
|  |  | 72010 | | Déficit vécu de soutien de la hiérarchie |
|  |  | 72011 | | Déficit vécu de soutien du collectif de travail ou des pairs |
|  | 7209 | | | Autres caractéristiques qualitatives des relations pouvant générer une nuisance |
| ***721*** | | | | ***Violence extérieure (personnes extérieures à l'entreprise/établissement)*** |
|  | 7210 | | | Agression verbale (*personnes extérieures à l'entreprise/établissement*) |
|  |  | 72010 | | Agression verbale sans menace crédible de mort (*personnes extérieures à l'entreprise/établissement*) |
|  |  | 72011 | | Agression verbale avec menace crédible de mort (*personnes extérieures à l'entreprise/établissement*) |
|  | 7211 | | | Agression physique |
|  | 7212 | | | Braquage, hold-up |
|  | 7213 | | | Etre témoin d'un événement traumatisant |
|  | 7219 | | | Autres violences extérieures |
| ***722*** | | | | ***Violence interne (personnes internes à l'entreprise/établissement)*** |
|  | 7220 | | | Agression verbale (*personnes internes à l'entreprise/établissement*) |
|  |  | 72200 | | Agression verbale sans menace crédible de mort (*personnes internes à l'entreprise/établissement*) |
|  |  | 72201 | | Agression verbale avec menace crédible de mort (*personnes internes à l'entreprise/établissement*) |
|  | 7221 | | | Fausse accusation |
|  |  | 72210 | | Fausse accusation sans procédure |
|  |  | 72211 | | Fausse accusation avec procédure |
|  | 7222 | | | Agression, violence physique subie |
|  | 7223 | | | Evénement traumatisant vécu comme témoin ou relaté |
|  |  | 72230 | | Etre témoin d’agression verbale, physique |
|  |  | 72231 | | Etre témoin de décès au travail hors suicide |
|  |  | 72232 | | Etre témoin de suicide au travail |
|  |  |  | 722320 | Etre témoin de suicide réussi au travail |
|  |  |  | 722321 | Etre témoin de tentative de suicide au travail |
|  |  | 72233 | | Relation d’agression verbale, physique ou de tentative de suicide |
|  |  |  | 722330 | Relation d’agression verbale, physique ou de tentative de suicide liée au travail, hors les lieux de travail (*menaces sur le trajet*) |
|  |  |  | 722331 | Relation d’agression verbale, physique ou de tentative de suicide survenue sur les lieux de travail |
|  |  | 72234 | | Relation de suicide réussi |
|  |  |  | 722340 | Relation de suicide réussi lié au travail hors les lieux de travail |
|  |  |  | 722341 | Relation de suicide réussi sur les lieux de travail |
|  |  | 72235 | | Relation de décès (*hors suicide*) lié au travail (*collègues*) quelque soit le lieu de survenue |
|  |  | 72239 | | Autres évènements traumatisants en relation avec le travail |
|  | 7224 | | | Vécu de harcèlement à caractère sexuel |
|  | 7225 | | | Vécu de discriminations (*genre, âge, préférence sexuelle, etc*.) |
|  | 7226 | | | Vécu de harcèlement moral au travail |
|  | 7227 | | | Déqualification |
|  | 7228 | | | Mise au placard |
|  | 7229 | | | Autres violences internes |
| ***729*** | | | | ***Autres modalités générales des relations au travail pouvant générer une nuisance*** |
| **73** | | | | **Ethique personnelle – conflit de valeur** |
| ***730*** | | | | ***Faire des actes allant à l'encontre de ses principes (vente abusive, réaliser des licenciements)*** |
| ***731*** | | | | ***Etre témoin impuissant d'actes allant à l'encontre de ses principes*** |
| ***732*** | | | | ***Manquer de moyens ou de temps pour faire un travail de qualité*** |
| ***739*** | | | | ***Autres conflits de valeur du fait de l’éthique personnelle*** |
| **74** | | | | **Ethique de l'entreprise** |
| ***740*** | | | | ***Niveau général de sécurité ou culture de sécurité faible*** |
| ***741*** | | | | ***Niveau général d'hygiène ou culture d'hygiène faible*** |
| ***742*** | | | | ***Manque de moyens de protection*** |
|  | 7420 | | | Manque de moyens de protection collective |
|  | 7421 | | | Manque de moyens de protection individuelle |
| ***743*** | | | | ***Manque de respect dans la communication verbale*** |
| ***749*** | | | | ***Autres manques à l'éthique de l'entreprise pouvant générer une nuisance*** |
| **75** | | | | **Autres facteurs contributeurs** |
| ***750*** | | | | ***Statut médical ou social particulier pouvant modifier les relations*** |
|  | 7500 | | | Retour après une interruption de travail pour maladie (*à l'exception du retour de congé maternité ou d'un AT/MP*) |
|  | 7501 | | | Prise en compte insuffisante ou inadaptée de restrictions d'aptitude (*hors* *handicap*) |
|  | 7502 | | | Retour après une absence |
|  |  | 75020 | | Retour après un AT/MP |
|  |  | 75021 | | Retour après un congé de maternité |
|  |  | 75022 | | Retour après un congé parental |
|  |  | 75023 | | Retour après un congé annuel |
|  |  | 75024 | | Retour après un CIF (congé individuel de formation) |
|  |  | 75025 | | Personne reconnue en situation de handicap |
|  | 7503 | | | Person recognized as having a disability |
|  | 7509 | | | Other medical or social status able to alter relations |
| ***751*** | | | | **Revendication de droits : concernant les congés, une formation, une prime non reçue, le paiement d'heures supplémentaires, signature d'une pétition** |
|  | 7510 | | | Actions conduites du fait d'un engagement social ou d'un mandat électif |
|  | 7511 | | | Actions conduites à titre personnel |
|  | 7519 | | | Autres modalités de revendication de droits |
| ***752*** | | | | ***Prise de position ou acte individuel mettant en cause l'entreprise*** |
|  | 7520 | | | Dénonciation d'agissements supposés ou prétendus malhonnêtes en rapport avec l'activité professionnelle |
|  | 7521 | | | Externalisation d'un problème interne à l'entreprise (*auprès de l'Inspection du Travail, d'un avocat*…) |
|  | 7529 | | | Autres prises de position ou acte individuel mettant en cause l'entreprise |
| ***753*** | | | | ***Modalités particulières choisies de travail*** |
|  | 7530 | | | Employeurs multiples |
|  | 7531 | | | Télétravail choisi |
|  | 7532 | | | Travail à domicile choisi |
|  | 7533 | | | Trajet domicile – travail |
|  |  | 75330 | | Trajet domicile – travail > 2h/j |
|  |  | 75331 | | Trajet domicile – travail > 3h/j |
|  | 7534 | | | Travail surqualifié |
|  | 7535 | | | Réaffectation souhaitée sur un autre poste |
|  |  | 75350 | | Réaffectation souhaitée sur un autre poste du même site |
|  |  | 75351 | | Réaffectation souhaitée sur un autre poste d’un autre site |
|  | 7536 | | | Poste ne correspondant pas aux attentes mais accepté pour raisons économiques |
|  | 7537 | | | Travail à temps partiel choisi |
|  | 7538 | | | Travail temporaire choisi |
|  | 7539 | | | Autres modalités particulières de travail choisies pouvant générer une nuisance |
| ***754*** | | | | ***Facteurs de majoration liés à l'entreprise (Facteurs social - Facteurs économique)*** |
|  | 7540 | | | Facteurs socio-économiques défavorables |
|  |  | 75400 | | Facteurs sociaux défavorables *(mouvements sociaux, grève-préavis)* |
|  |  | 75401 | | Facteurs économiques défavorables *(chômage technique, blocage des salaires, difficultés financières de l'entreprise)* |
|  |  | 75409 | | Autres facteurs socio-économiques défavorables |
|  | 7541 | | | Procédure de rupture conventionnelle de contrat ou négociation de départ volontaire en cours (ne coder que si vécu défavorablement) |
|  | 7542 | | | Insécurité dans l'emploi |
|  | 7543 | | | Engagement d’une procédure de licenciement |
|  |  | 75430 | | Engagement d’une procédure de licenciement économique |
|  |  | 75431 | | Engagement d’une procédure de licenciement individuelle |
|  | 7544 | | | Facteurs d’entreprise familiale ou de liens particuliers entre la personne et la hiérarchie |
|  | 7549 | | | Autres Facteurs défavorables liés à l'entreprise |
| ***759*** | | | | ***Autres modalités générales constituant facteur de majoration*** |
