# Supplementary material for: Occupational exposure factors for mental and behavioral disorders at work: The FOREC thesaurus
Source: PLoS One. 2018 Jun 21;13(6):e0198719. doi: 10.1371/journal.pone.0198719 (PMC6013225; doi:10.1371/journal.pone.0198719)
Supplement: S1 Table — (DOCX) [file pone.0198719.s003.docx]

**Table .** Number of workers per occupational group and per level of function according the ISCO-08 classification (n=322 because of one missing value)

|  |  | **n=322** |
| --- | --- | --- |
| **1 – Managers** | | **39 (12.1)** |
|  | 12 Administrative and Commercial Managers | 19 (5.9) |
|  | 13 Production and Specialized Services Managers | 15 (4.7) |
|  | 14 Hospitality, Retail and Other Services Managers | 5 (1.5) |
| **2 – Professionals** | | **72 (22.4)** |
|  | 21 Science and Engineering Professionals | 6 (1.9) |
|  | 22 Health Professionals | 19 (5.9) |
|  | 23 Teaching Professionals | 10 (3.1) |
|  | 24 Business and Administration Professionals | 14 (4.3) |
|  | 25 Information and Communications Technology Professionals | 6 (1.9) |
|  | 26 Legal, Social and Cultural Professionals | 17 (5.3) |
| **3 – Technicians and Associate Professionals** | | **76 (23.6)** |
|  | 31 Science and Engineering Associate Professionals | 9 (2.8) |
|  | 32 Health Associate Professionals | 17 (5.3) |
|  | 33 Business and Administration Associate Professionals | 40 (12.4) |
|  | 34 Legal, Social, Cultural and Related Associate Professionals | 10 (3.1) |
| **4 – Clerical Support Workers** | | **47 (14.6)** |
|  | 41 General and Keyboard Clerks | 30 (9.3) |
|  | 42 Customer Services Clerks | 9 (2.8) |
|  | 43 Numerical and Material Recording Clerks | 6 (1.9) |
|  | 44 Other Clerical Support Workers | 2 (0.6) |
| **5 – Services and Sales Workers** | | **44 (13.7)** |
|  | 51 Personal Services Workers | 12 (3.7) |
|  | 52 Sales Workers | 15 (4.7) |
|  | 53 Personal Care Workers | 6 (1.9) |
|  | 54 Protective Services Workers | 11 (3.4) |
| **6 – Skilled Agricultural, Forestry and Fishery Workers** | | **4 (1.2)** |
|  | 61 Market-oriented Skilled Agricultural Workers | 4 (1.2) |
| **7 – Craft and Related Trades Workers** | | **9 (2.8)** |
|  | 71 Building and Related Trades Workers (excluding electricians) | 3 (0.9) |
|  | 72 Metal, Machinery and Related Trades Workers | 3 (0.9) |
|  | 75 Food Processing, Woodworking, Garment and Other Craft and Related Trades Workers | 3 (0.9) |
| **8 – Plant and Machine Operators and Assemblers** | | **4 (1.2)** |
|  | 83 Drivers and Mobile Plant Operators | 4 (1.2) |
| **9 – Elementary Occupations** | | **27 (8.4)** |
|  | 91 Cleaners and Helpers | 17 (5.3) |
|  | 93 Labourers in Mining, Construction, Manufacturing and Transport | 3 (0.9) |
|  | 94 Food Preparation Assistants | 1 (0.3) |
|  | 96 Refuse Workers and Other Elementary Workers | 6 (1.9) |

*Data are presented as frequencies (associated percentages).*
